# Clinician Perceptions of Family-Centered Care in Pediatric and Congenital Heart Settings

**DOI:** 10.1001/jamanetworkopen.2024.22104

**Published:** 2024-07-15

**Authors:** Farah Akram, Annabel E. Webb, Madeleine Pidcock, Michelle A. Farrar, Nadine A. Kasparian

**Affiliations:** 1 Heart Centre for Children, The Sydney Children’s Hospitals Network, Sydney, Australia; 2 Discipline of Paediatrics and Child Health, School of Clinical Medicine, UNSW Medicine and Health, UNSW Sydney, Sydney, Australia; 3 Discipline of Child and Adolescent Health, Faculty of Health and Medicine, The University of Sydney, Sydney, Australia; 4 School of Mathematical and Physical Sciences, Faculty of Science and Engineering, Macquarie University, Sydney, Australia; 5 Heart and Mind Wellbeing Center, Heart Institute and the Division of Behavioral Medicine and Clinical Psychology, Cincinnati Children’s Hospital Medical Center, Cincinnati, Ohio; 6 Department of Pediatrics, University of Cincinnati College of Medicine, Cincinnati, Ohio

## Abstract

**Importance:**

Family-centered care recognizes families as central to child health and well-being and prioritizes clinician collaboration with families to ensure optimal pediatric care and outcomes. Clinician interpersonal sensitivity and communication skills are key to this approach.

**Objective:**

To examine perceptions of and factors associated with family-centered care among clinicians working in pediatric and congenital heart care.

**Design, Setting, and Participants:**

In this cross-sectional study, participants from diverse clinical disciplines (pediatric cardiology, cardiothoracic surgery, nursing, anesthesia, neonatology, intensive care, psychology, and others), completed an online survey between June 2020 and February 2021. Participants included physicians, surgeons, nurses, and allied and mental health professionals at an Australian quaternary pediatric hospital network. Statistical analysis was performed from August 2022 to June 2023.

**Main Outcomes and Measures:**

Family-centered care across 4 domains (showing interpersonal sensitivity, treating people respectfully, providing general information, and communicating specific information) was measured using the validated Measure of Processes of Care for Service Providers. Clinician burnout (emotional exhaustion, depersonalization, and personal accomplishment), confidence responding to families’ psychosocial needs, and psychological, clinical role, and sociodemographic factors were also assessed. Informed by theory, hierarchical linear regression was used to identify factors associated with family-centered care.

**Results:**

There were 212 clinicians (177 women [84.3%]; 153 nurses [72.2%], 32 physicians [15.1%], 22 allied and mental health professionals [10.4%], 5 surgeons [2.3%]; 170 [80.2%] aged 20-49 years) who participated (55% response rate). Of the 4 family-centered care domains, scores for treating people respectfully were highest and associated with greater clinician confidence responding to families’ psychosocial needs (effect size [β], 0.59 [95% CI, 0.46 to 0.72]; *P* < .001), lower depersonalization (β, 0.04 [95% CI, −0.07 to −0.01]; *P* = .02), and a greater sense of personal accomplishment at work (β, 0.02 [95% CI, 0.01 to 0.04]; *P* = 0.04). Greater interpersonal sensitivity was associated with greater confidence responding to families’ psychosocial needs (β, 0.80 [95% CI, 0.62 to 0.97]; *P* < .001), a greater sense of personal accomplishment at work (β, 0.03 [95% CI, 0.01 to 0.05]; *P* = .04), and lower use of approach-based coping, such as problem-solving (β, 0.37 [95% CI, −0.71 to −0.02]; *P* = .04).

**Conclusions and Relevance:**

In this cross-sectional study, burnout and confidence responding to families’ psychosocial needs were associated with clinicians’ perceptions of family-centered care. These findings suggest that targeted interventions to address these factors may benefit clinicians and also potentially strengthen the practice of family-centered care in pediatric and congenital heart settings.

## Key Points

**Question:**

What factors are associated with clinicians’ ratings of family-centered care in congenital heart centers?

**Findings:**

In this cross-sectional study of 212 clinicians across diverse roles, confidence responding to families’ psychosocial needs was associated with clinicians’ perceptions of family-centered care. A greater sense of personal accomplishment, a key factor in assessing burnout, was associated with higher scores for treating people respectfully and interpersonal sensitivity.

**Meaning:**

These findings suggest that improving clinician confidence and well-being may benefit clinicians and also strengthen family-centered care in congenital heart settings.

## Teaser

This cross-sectional study investigates perceptions of and factors associated with family-centered care among clinicians working in pediatric and congenital heart care.

## Introduction

Family-centered care involves integration of the family in the health care of children to optimize health outcomes.^1^ This approach includes bidirectional communication of information between clinicians and family members, shared decision-making, and continuity of care for children and families through all stages of disease management.^2,3^ Family-centered care is associated with improved outcomes for patients, including fewer emergency department visits,^4^ and greater quality of life,^5^ as well as improved parent and family well-being,^6^ better understanding of their child’s health condition and treatment options,^7^ increased uptake of appropriate health services,^8^ and greater satisfaction with clinical care.^9^

For clinicians, successful delivery of family-centered care is associated with improved communication and collaboration between colleagues^10^ and with families,^11^ as well as greater workplace morale^12^ and work engagement.^13^ Despite the many demonstrated benefits, family-centered care can be challenging. On an individual level, family-centered care may require skills that extend beyond clinicians’ expertise, such as identifying and responding to families’ psychosocial needs.^14^ Clinician burnout and moral distress can adversely affect clinical care,^15,16^ as can workplace factors, such as workload, staffing, team coordination, and access to appropriate support services for patients and families.^13,17,18,19^

Clinicians in pediatric and congenital heart centers work in high acuity, resource-intensive environments involving collaboration with multidisciplinary teams and highly distressed patients and families.^20,21,22^ Studies exploring communication between physicians and parents of children with heart disease report low physician-parent agreement regarding disease prognosis and child quality of life, with parents often rating communication as inadequate or inconsistent.^23,24,25,26^ Pediatric cardiology fellows and cardiac nurses perceive caring for sick babies and distressed parents as a challenging part of their job,^27,28^ especially in the context of high workload.^27,28,29,30^ Thus, the aims of this study were 2-fold: (1) to explore self-reported family-centered care practices and associated factors among clinicians working in pediatric and congenital heart settings; and (2) assess clinicians’ confidence in responding to the psychosocial needs of patients and families affected by congenital heart disease (CHD). We hypothesized that clinicians who reported greater burnout (including emotional exhaustion, depersonalization, and low personal accomplishment) would report lower family-centered care.^31^

## Methods

### Study Design and Participants

This cross-sectional study followed the Strengthening the Reporting of Observational Studies in Epidemiology (STROBE) reporting guideline. Participants were recruited from a large Australian quaternary hospital network, including the Children’s Hospital at Westmead and the Sydney Children’s Hospital at Randwick. Clinicians from diverse disciplines involved in caring for children with heart disease, who were contactable at the time of recruitment, were invited to complete 1 survey. Approval was granted by the relevant Human Research Ethics Committee (HREC/15/SCHN/95).

### Recruitment

Recruitment opened in June 2020 and closed in February 2021. Potential participants were identified via their hospital departments. A personalized invitation, detailed study information sheet, and customized link to the online survey hosted on the secure Qualtrics platform (Qualtrics) were sent to all eligible clinicians via email. A paper-based survey was provided to those who had not completed the online survey after 3 reminders. With support from department directors and team leaders, the survey was also introduced through information sessions which provided a brief overview of the study, consistent with the study information sheet. Recruitment strategies included distribution of customized cookies and chocolates, study flyers, and digital posters. Survey completion indicated informed consent.

### Measures

The WithCare Health Professional Survey included validated and study-specific measures, selected based on a thorough literature review and the National Academy of Medicine Conceptual Model for Clinician Well-Being and Resilience.^32^ The survey was tested in 5 rounds of piloting with clinicians from psychology, pediatric cardiology, cardiac nursing, anesthesia, and social work, with feedback from each round incorporated to improve survey flow, optimize study engagement, and reduce participant burden.

The primary outcome, self-reported practice of family-centered care, was measured using the validated and widely used Measure of Processes of Care for Service Providers (MPOC-SP; 27 items),^33^ which assesses pediatric clinicians’ perceptions of the extent to which they provide family-centered care across 4 domains: showing interpersonal sensitivity (10 items; Cronbach α in the present study = 0.94), treating people respectfully (9 items; α = 0.93), communicating specific information (3 items; α = 0.85), and providing general information (5 items, α = 0.92). Statements about interactions with patients and families in the past 12 months are rated on a scale from 1 (not at all) to 7 (a very great extent), with 0 selected if the situation does not apply. A mean score is calculated for each domain, with higher scores indicating greater family-centered care.

#### Sociodemographic Characteristics (8 Items)

Participant gender, age, educational attainment, marital status, gross annual household income, birth country, language primarily spoken at home, and number of children were assessed using standard items. Household income was compared with the Australian national average of A $120 000 (US $85 700) per annum.^34^

#### Clinical Role (7 Items)

Assessed primary clinical role, years of CHD-specific experience, work hours per week, days on call in the past month, shift work, work area, and employment status (permanent, fixed-term, casual). Work area was categorized as intensive (including pediatric and neonatal intensive care units and operating suite), or nonintensive (including cardiac ward, outpatient clinic, catheterization laboratory, office).

#### Burnout (22 Items)

Three well-established burnout dimensions were assessed using the validated, widely used Maslach Burnout Inventory (MBI): emotional exhaustion (9 items; α = 0.91), depersonalization (5 items; α = 0.78), and personal accomplishment at work (8 items; α = 0.77).^35^ Participants rated statements regarding job-related experiences over the past year from 0 (never) to 6 (every day). Percentage of endorsement of key items and mean scores for each dimension were calculated, with higher scores for emotional exhaustion and depersonalization, and lower scores for personal accomplishment, indicating greater burnout.

#### Empathy Toward Patients (20 Items)

Using the validated Jefferson Scale of Empathy (20 items; α = 0.81),^36^ participants indicated agreement with 20 statements from 1 (strongly disagree) to 7 (strongly agree). After reverse scoring of specific items, a total score was computed, with higher scores indicating greater empathy in health care.

#### Resilience (6 Items)

Resilience was assessed using the validated Brief Resilience Scale (6 items; α = 0.82).^37^ Participants indicated agreement with 6 statements on a scale from 1 (strongly disagree) to 5 (strongly agree), with higher scores indicating greater psychological resilience.

#### Coping (18 Items)

Nine subscales of the Brief COPE (18 items)^38^ were used to assess the frequency of coping responses to work-related difficulties, with responses ranging from 1 (“I don’t do this at all”) to 4 (“I do this a lot”). Two mean scores were calculated using the Eisenberg classification for coping styles^39^: (1) approach-based coping (α = 0.69), including active coping, positive reframing, and acceptance, and (2) avoidance-based coping (α = 0.71), including self-distraction, denial, substance use, behavioral disengagement, venting, and self-blame.

#### Perceived Social Support (5 Items)

Using the validated Social Provisions Scale (SPS-5) (5 items; α = 0.87),^40^ participants rated statements about the extent to which they felt supported in their current personal and professional relationships from 1 (strongly disagree) to 4 (strongly agree), with higher scores indicating greater perceived social support.

#### Work-Family Conflict (5 Items)

Using the validated Work-Family Conflict scale (5 items; α = 0.95),^41^ participants rated statements from 1 (strongly disagree) to 7 (strongly agree) to reflect the extent to which work disrupted family life and vice versa. Higher scores indicate greater conflict between work and family life.

#### Anxiety and Depressive Symptoms (4 Items)

Using the Patient Health Questionnaire (PHQ-4) (4 items; α = 0.84), participants indicated the extent to which they experienced anxiety and depressive symptoms in the past 2 weeks from 0 (not at all) to 3 (nearly every day), with higher total scores for each subscale indicating greater symptoms. Scores of at least 3 on either subscale (anxiety, depression) indicate a need for clinical intervention and participants were contacted by a psychologist to provide support options.

#### Confidence Responding to Families’ Psychosocial Needs (9 Items)

A purpose-designed measure was developed to assess clinicians’ confidence responding to the psychosocial needs of patients and families (eg, talking with families about child neurodevelopmental outcomes, referring a child or parent to a psychologist). Responses ranged from 1 (not at all confident) to 5 (extremely confident), with 0 selected if the item was not applicable, and higher mean scores indicated greater confidence. This measure had excellent internal consistency (α = 0.90).

#### Mental Health Knowledge (7 Items)

Mental health knowledge wa assessed using a study-specific measure comprising 7 multiple-choice questions, each with 5 response options, including “I don’t know” (eg, “According to Australian research, what proportion of parents meet clinical criteria for depression within one year after their baby’s diagnosis of complex congenital heart disease?”). Correct items were summed to yield a total score out of 7, with higher scores indicating greater mental health–related knowledge.

### Statistical Analysis

Statistical analyses were performed using SPSS Statistics version 27.0 (IBM) or R version 4.1.3 (R Project for Statistical Computing) from August 2022 to June 2023. Participants who completed less than 60% of the survey were excluded from analysis. Variables with missing data were computed as per instrument manuals. χ^2^ tests of association were used to compare characteristics of study participants and eligible nonparticipants (eg, work role). Descriptive statistics (eg, mean [SD]) were used to explore sociodemographic, clinical role, and psychosocial characteristics. Differences in mean scores for each of the MPOC-SP domains were tested for statistical significance using a repeated measures analysis of variance (ANOVA) with a Greenhouse-Geisser correction.^42^ A paired samples *t* test was used to compare mean scores of the 2 coping subscales, while differences in mean confidence scores for each clinical group were tested for significance using ANOVA.

Hierarchical linear regression models were built to examine correlates of 3 domains of family-centered care (showing interpersonal sensitivity, treating people respectfully, and communicating specific information). The MPOC-SP domain of providing general information was not included as this assessed organizational rather than clinician-specific attributes. A power calculation was carried out, assuming a moderate effect size (*F^2^* = 0.15), with a 5% significance level and 80% power for 20 variables, giving a total required sample size (N) of 157. Selection of correlates in the regression model was based on theory and published evidence. Correlates were grouped into 4 blocks and entered into each model in the following order: (1) sociodemographics, (2) clinical role characteristics, (3) psychosocial factors, and (4) clinician confidence responding to families’ psychosocial needs. Multicollinearity among correlates was checked by computing the variance inflation factor (VIF) for each model after addition of each block of correlates. Assumptions of normally distributed residuals and homogeneity of residual variance required for linear regression were checked for each model using residual plots. While there was some collinearity between years of CHD-specific experience and age, anxiety and depression, and anxiety and emotional exhaustion, no included correlate had a VIF greater than 5 in each final model. The proportion of variance in self-reported family-centered care explained by each block was evaluated by computing the change in *R^2^* for each model. All tests and confidence intervals were 2-sided, and statistical significance was defined as *P* < .05.

## Results

### Response Rate and Sample Sociodemographic Characteristics

Among 212 clinicians who participated (response rate: 55% [212 of 387]), 153 (72.2%) were nurses, 32 (15.1%) were physicians, 22 (10.4%) were allied and mental health professionals, and 5 (2.3%) were surgeons; 177 (84.3%) were women, 170 (80.2%) were aged 20 to 49 years, 146 (70.4%) were married or in a relationship, and 149 (70.9%) were Australian-born (Table 1). The mean (SD) length of experience working in pediatric cardiac care was 8.4 (7.8) years, ranging from 6 months to 38 years; 141 participants (66.5%) worked in intensive care areas, and 188 (88.7%) had permanent employment. There were no differences between participants and eligible nonparticipants in terms of primary clinical role or gender. While health professionals working in intensive areas (response rate: 47% [141 of 297]) comprised more than 75% of the eligible cohort, those working in nonintensive areas were more likely to complete the survey (response rate: 78% [71 of 90]; χ^2^_1_ = 16.9; *P* < .001).

**Table 1.** Sample Sociodemographic and Clinical Role Characteristics (N = 212)^a^

Variable	Total sample, No. (%)
Gender	
Women	177 (84.3)
Men	29 (13.8)
Prefer not to say	4 (2.0)
Age, y	
20-29	59 (27.6)
30-39	63 (29.8)
40-49	48 (22.4)
50-59	26 (12.3)
≥60	10 (4.4)
Prefer not to answer	2 (0.4)
Educational attainment	
No university degree	13 (6.1)
Undergraduate degree	61 (28.5)
Postgraduate degree	132 (62.2)
Country of birth	
Australia	149 (70.9)
Overseas	59 (27.1)
Language primarily spoken at home	
English	183 (87.6)
Other	26 (12.4)
Marital status	
Never married	49 (23.5)
Married or partnered	146 (70.4)
Divorced or separated	14 (6.1)
Children	
Yes	107 (51.7)
No	100 (48.3)
Gross annual household income	
Below national average	85 (39.3)
Above national average	95 (44.3)
Prefer not to answer	32 (14.5)
Primary clinical role	
Physician or surgeon^b^	37 (17.5)
Consultant	26 (12.3)
Fellow	8 (3.7)
Registrar	3 (1.4)
Nurse	153 (72.2)
Allied or mental health professional	22 (10.4)
Professional experience, mean (SD), y	11.7 (10.0)
Experience working in CHD, mean (SD), y	8.4 (7.8)
Hours worked per week, mean (SD)	38.1 (9.2)
Area of work based on clinical intensity	
Urgent, intensive care	141 (66.5)
Nonurgent, nonintensive care	71 (33.5)
Employment status	
Permanent	188 (88.7)
Fixed-term contract	21 (9.9)
Casual, locum, or leave relief	2 (0.9)
Other	1 (0.5)
Night shifts	
Yes	25 (11.8)
No	187 (88.2)

^a^ Some percentages do not add to 100 due to missing data.

^b^ Specialties: fetal or pediatric cardiology (n = 11), neonatology (n = 7), pediatric cardiothoracic surgery (n = 5), pediatric intensive care (n = 6), and anesthesiology (n = 7).

### Psychosocial Characteristics

In the past month, 141 participants (67%) reported at least 1 symptom of emotional exhaustion, 62 (30%) reported at least 1 symptom of depersonalization, and 170 (80%) endorsed at least 1 area of personal accomplishment at work. There were 36 (17%) who reported anxiety symptoms and 21 (10%) who reported depressive symptoms warranting clinical assessment. Participants reported using approach-based coping (eg, positive reframing) more frequently than avoidance-based coping (eg, denial: *t* = −29.2; *df* = 211; *P* < .001). While perceived social support was high, so was work-family conflict (Table 2).

**Table 2.** Self-Reported Practice of Family-Centered Care, Psychosocial Characteristics, and Confidence Responding to Families’ Psychosocial Needs (N = 212)

Variable	Score, points	
	Mean (SD)	Possible range
Family-centered care		
Showing interpersonal sensitivity	4.9 (1.0)	1-7
Treating people respectfully	5.5 (0.8)	1-7
Communicating specific information	4.3 (1.4)	1-7
Providing general information	4.3 (1.4)	1-7
Burnout		
Emotional exhaustion	21.4 (11.2)	0-54
Depersonalization	4.6 (4.6)	0-30
Personal Accomplishment	35.7 (7.1)	0-48
Empathy	110.4 (12.6)	20-140
Anxiety	1.5 (1.6)	0-6
Depressive symptoms	1.0 (1.4)	0-6
Coping		
Approach-based coping	3.0 (0.5)	1-4
Avoidance-based coping	1.9 (0.4)	1-4
Psychological resilience	3.5 (0.6)	1-5
Perceived social support	17.7 (2.5)	5-20
Work-family conflict	20.9 (8.1)	5-35
Confidence responding to families’ psychosocial needs		
Physicians and surgeons	3.1 (0.8)	1-5
Nurses	2.8 (0.9)	1-5
Allied and mental health professionals	3.0 (1.1)	1-5

### Family-Centered Care and Confidence Responding to Families’ Psychosocial Needs

Of the 4 domains of family-centered care, the mean (SD) score for treating people respectfully was higher than other domains (5.5 [0.9]; *F* = 78.5; *df* = 2; *P* < .001), followed by showing interpersonal sensitivity (4.9 [1.0]) (Table 2). Communicating specific information (mean [SD] score, 4.3 [1.3]) and providing general information (mean [SD] score, 4.3 [1.4]) were rated lowest, with no difference in mean scores. Differences in confidence responding to families’ psychosocial needs among physicians (mean [SD] score, 3.1 [0.8]), allied and mental health professionals (mean [SD] score, 3.0 [1.1]), and nurses (mean [SD] score, 2.8 [0.9]) did not reach statistical significance.

### Factors Associated With Family-Centered Care

Three hierarchical regression models were developed to identify factors associated with self-reported practice of family-centered care (Table 3). Factors associated with showing interpersonal sensitivity (model 1) included greater confidence responding to families’ psychosocial needs (β, 0.80 [95% CI, 0.62-0.97]; *P* < .001), greater personal accomplishment at work (β, 0.03 [95% CI, 0.01-0.05]; *P* = .04), and lower use of approach-based coping (β, −0.37 [95% CI, −0.71 to −0.02]; *P* = .03), with the model accounting for 47% of the variance in interpersonal sensitivity. Factors associated with treating people respectfully (model 2) included greater personal accomplishment at work (β, 0.02 [95% CI, 0.01-0.04]; *P* = .04), greater confidence responding to families’ psychosocial needs (β, 0.59 [95% CI, 0.46-0.72]; *P* < .001), and lower depersonalization (β, −0.04 [95% CI, −0.07 to −0.01]; *P* = .02), with 50% of variance explained by this model. For communicating specific information (model 3), higher scores were reported by physicians (β, 0.84 [95% CI, 0.06 to 1.61]; *P* = 0.03, compared with the reference category of nurses), clinicians with a postgraduate degree (β, 0.48 [95% CI, 0.01-0.94]; *P* = .04), and those with greater confidence responding to families’ psychosocial needs (β, 0.82 [95% CI, 0.55-1.09]; *P* < .001), with 36% of variance explained by this model.

**Table 3.** Hierarchal Regression Models to Identify Correlates of 3 Domains of Family-Centered Care

Variable	β (95% CI)		
	Model 1: Showing interpersonal senstivity	Model 2: Treating people respectfully	Model 3: Communicating specific information
Block 1: sociodemographic factors			
Gender			
Men	[Reference]	[Reference]	[Reference]
Women	0.23 (−0.25 to 0.70)	−0.06 (−0.43 to 0.32)	−0.40 (−1.11 to 0.31)
Age			
<30 y	0.25 (−0.10 to 0.59)	0.07 (−0.19 to 0.34)	0.34 (−0.20 to 0.89)
30-50 y	[Reference]	[Reference]	[Reference]
>50 y	−0.11 (−0.50 to 0.28)	−0.06 (−0.37 to 0.24)	−0.30 (−0.89 to 0.30)
Education level			
Undergraduate or below	[Reference]	[Reference]	[Reference]
Postgraduate	0.09 (−0.20 to 0.39)	0.14 (−0.10 to 0.37)	0.89 (0.01 to 0.94)
Language spoken at home			
English	[Reference]	[Reference]	[Reference]
Other	0.10 (−0.35 to 0.55)	0.00 (−0.33 to 0.33)	−0.04 (−0.70 to 0.61)
Marital status			
Married/partnered	[Reference]	[Reference]	[Reference]
Not married/partnered	0.14 (−0.18 to 0.46)	0.08 (−0.16 to 0.32)	0.02 (−0.47 to 0.52)
*R* ^2^	0.03	0.03	0.11
*F* statistic	0.84	0.84	3.16
*P* value	.54	.54	<.001
Block 2: clinical role characteristics			
Primary clinical role			
Nurse	[Reference]	[Reference]	[Reference]
Medical doctor	0.10 (−0.42 to 0.63)	−0.31 (−0.70 to 0.09)	0.84 (0.06 to 1.61)
Mental and allied health professional	−0.05 (−0.54 to 0.44)	−0.02 (−0.38 to 0.33)	0.07 (−0.77 to 0.91)
Years of experience in congenital heart disease care	0.01 (−0.01 to 0.03)	0.00 (−0.02 to 0.02)	0.01 (−0.01 to 0.05)
Total hours worked per week	0.00 (−0.02 to 0.02)	−0.010 (−0.02 to 0.01)	−0.02 (−0.02 to 0.05)
Clinical intensity of work area			
Nonintensive care	[Reference]	[Reference]	[Reference]
Intensive care	0.03 (−0.27 to 0.32)	0.07 (−0.16 to 0.30)	−0.13 (−0.59 to 0.32)
*R* ^2^	0.07	0.06	0.14
Change in *R*^2^	0.04	0.03	0.03
*F* statistic	1.39	1.10	1.15
*P* value	.23	.36	.34
Block 3: psychosocial factors			
Empathy	0.00 (−0.01 to 0.01)	0.01 (−0.01 to 0.03)	0.00 (−0.02 to 0.02)
Emotional exhaustion	0.00 (−0.02 to 0.02)	0.00 (−0.01 to 0.01)	0.01 (−0.02 to 0.04)
Depersonalization	0.00 (−0.04 to 0.04)	−0.04 (−0.07 to −0.01)	−0.01 (−0.07 to 0.05)
Personal accomplishment at work	0.03 (0.01 to 0.05)	0.02 (0.01 to 0.04)	0.02 (−0.02 to 0.06)
Anxiety symptoms	0.05 (−0.05 to 0.16)	0.01 (−0.08 to 0.10)	−0.04 (−0.21 to 0.13)
Depressive symptoms	−0.03 (−0.16 to 0.09)	0.02 (−0.08 to 0.12)	0.03 (−0.17 to 0.23)
Use of approach coping	−0.37 (−0.71 to −0.02)	−0.24 (−0.50 to 0.01)	−0.35 (−0.86 to 0.17)
Use of avoidant coping	0.23 (−0.23 to 0.68)	0.17 (−0.18 to 0.53)	0.39 (−0.31 to 1.08)
Perceived social support	−0.00 (−0.06 to 0.06)	−0.02 (−0.07 to 0.03)	−0.08 (−0.17 to 0.01)
Work-family conflict	−0.02 (−0.04 to 0.01)	0.00 (−0.02 to 0.02)	−0.02 (−0.05 to 0.01)
*R* ^2^	0.19	0.25	0.20
Change in *R*^2^	0.12	0.19	0.06
*F* statistic	2.10	3.90	0.92
*P* value	.03	<.001	.52
Block 4: Confidence			
Confidence responding to families’ psychosocial needs	0.80 (0.62 to 0.97)	0.59 (0.46 to 0.72)	0.82 (0.55 to 1.09)
*R* ^2^	0.47	0.50	0.36
Change in *R*^2^	0.28	0.25	0.16
*F* statistic	78.21	77.39	34.00
*P* value	<.001	<.001	<.001

### Mental Health Knowledge

Overall, out of 7 total points possible, the mean (SD) mental health knowledge score for the sample was 2.35 (1.56) points. Only 3 participants (1%) answered all 7 questions correctly. While 147 participants (69%) were able to identify symptoms of posttraumatic stress, less than one-quarter of clinicians correctly answered questions about the prevalence of postpartum depression in Australian women and men (Table 4).

**Table 4.** Clinician Knowledge of Mental Health (N = 212)

Item	Question	Participants answering correctly, No. (%)
1	What percentage of Australian women experience the ‘postpartum blues’?	47 (22)
2	According to Australian research, how many women experience symptoms of depression warranting clinical intervention in the first year after giving birth?	101 (48)
3	What percentage of men experience postnatal depression?	47 (22)
4	According to Australian research, what proportion of parents meet clinical criteria for depression within 1 year of their baby’s diagnosis of complex congenital heart disease (CHD)?	58 (27)
5	A diagnosis of posttraumatic stress disorder (PTSD) requires the presence of symptoms in 4 key domains. Which is NOT one of these domains?	147 (69)
6	Infants who have had cardiac surgery in the first 3 mos of life experience the greatest neurodevelopmental difficulties in which domain?	52 (25)
7	Adverse childhood experiences (ACES) can include abuse, neglect, and exposure to domestic violence, parental mental illness, or incarceration. Compared with children who have not experienced any ACEs, children who have been exposed to 5 or more ACEs are how much more likely to attempt suicide at some time in their life?	46 (21)

## Discussion

A family-centered approach is integral to providing high-quality pediatric care and achieving optimal child health outcomes.^1,3,12^ The practice of family-centered care can be challenging in congenital heart settings due to the high medical acuity and intense psychological distress experienced by patients and families.^12,43,44^ In this study, Australian clinicians working in congenital heart settings generally perceived themselves as providing family-centered care a ‘moderate’ to ‘fairly great’ extent. Greater clinician confidence responding to families’ psychosocial needs and lower burnout were associated with greater self-reported family-centered care practice, supporting our hypothesis.

The negative association between depersonalization and treating people respectfully may manifest as clinician detachment from patients, an inclination to treat patients and families as generic cases rather than individuals with distinct needs, and decreased capacity to build partnerships with patients and families.^35,45,46,47^ Approach-based coping was associated with lower ratings of interpersonal sensitivity, highlighting how heavy workload, understaffing, and greater time on non–patient-facing tasks (eg, maintaining electronic health records) may cause clinicians to prioritize problem-solving over showing sensitivity to patients and families.^10,17^ In contrast, scores for treating people respectfully and showing interpersonal sensitivity were both positively associated with personal accomplishment at work. Literature shows that supportive, staff-focused environments with good team communication improve feelings of personal accomplishment and professional fulfillment for pediatric clinicians, which in turn increase parental satisfaction with care.^5,27,28,45,48^ Conversely, insufficient training, high workload, and lack of organizational support and resources, are often identified by clinicians as obstacles to implementing family-centered care practices.^14,49,50^ A 2017 meta-analysis exploring the efficacy of interventions targeting physician burnout found organizational interventions (n = 5 studies) targeting workload, organizational structures, and team resources were more effective in reducing burnout than individual-focused interventions, such as mindfulness, cognitive-behavioral therapy, and exercise.^51^ Fostering a positive workplace culture is critical to addressing burnout^51,52,53^ and may be achieved through increased work flexibility, a focus on team-building, and reduced administrative burden, as highlighted in the National Academy of Medicine’s plan for health-workforce well-being and burnout prevention.^46,48,54^

While clinicians’ confidence responding to families’ psychosocial needs was positively associated with all 3 domains of family-centered care; overall, clinicians’ confidence ratings were low, as was their mental health knowledge. This signals a need for further education and training to cultivate clinicians’ knowledge, confidence, and skills in these areas. Research shows clinicians frequently underestimate the incidence of anxiety and depressive disorders in parents of children with heart disease, with adverse effects on parental participation in clinical decision-making and family-centered care.^55,56,57^ Pediatric cardiac trainees and nurses transitioning to congenital heart settings have reported fears of patient harm and a perceived inability to manage the responses of distressed families due to inadequate specialty-focused knowledge, skills, and training.^27,28,30^ The broader literature highlights the benefits of simulation boot camps to increase clinical knowledge and skills as well as improve confidence in fellows and nurses transitioning to pediatric cardiac care.^58,59,60^ Similar programs to increase clinicians’ mental health knowledge and communication skills when responding to families’ psychosocial needs may improve provision of family-centered care in congenital heart settings.^61,62,63,64^ The Institute for Patient- and Family-Centered Care advocates for integration of evidence-based patient- and family-centered care content into medical education curriculum through a variety of methods, including didactic classroom education, and a strengths-based approach that maximizes use of individual, family, and community resources to improve health outcomes.^65,66,67^ It also encourages understanding the diverse experiences and perspectives of patients and families through storytelling,^66^ shadowing,^68^ simulation exercises,^63^ and family-centered rounds,^69^ which can promote empathetic communication, cultural sensitivity, and reflective practices among clinicians.

### Areas for Future Research

To build on these findings, an important next step would be to explore and compare patient and family perceptions of family-centered care in pediatric heart settings. A deeper understanding of clinical team processes and support needs may also improve multidisciplinary collaboration and family-centered care practices. Multicenter studies assessing clinicians’ mental health knowledge, skills, and confidence responding to families’ psychosocial needs will allow for comparability of results and highlight areas for education and training.

### Limitations

This study has limitations. The cross-sectional study design precluded causal inference. Recruitment of clinicians, predominantly nurses and women, from an Australian health network during the COVID-19 pandemic may limit the generalizability of results, given Australia implemented stricter measures, such as domestic and international travel restrictions, diligent contact tracing, and lockdowns to suppress local outbreaks, in addition to social distancing and mask-wearing during the early stages of the pandemic, resulting in an initial lower COVID-19 caseload relative to some other countries.^70,71^ Clinicians experiencing greater psychological stress or workload demands may not have responded to the survey, potentially leading to an underestimation of the difficulties and needs of this population. The MPOC-SP assesses clinicians’ perceptions of the extent to which their practice is family-centered rather than actual behaviors; however, mean scores of 4 (moderate extent) to 5 (fairly great extent) across the 4 domains suggests it is unlikely participants overestimated positive practices. The recruitment strategies and use of self-report measures could have led to response bias and socially desirable responding; however, the low scores for several measures (eg, confidence responding to families’ psychosocial needs) suggest bias was minimal.

## Conclusions

Family-centered care is a critical aspect of pediatric cardiac care and is associated with clinician burnout, coping, and confidence responding to families’ psychosocial needs. This study identified gaps in clinicians’ mental health knowledge and confidence, highlighting the need for strategies to improve clinician well-being, mental health knowledge, and confidence to not only benefit clinicians but to also strengthen family-centered care in congenital heart settings.

## Notes

Supplement.
Data Sharing Statement
