# Supplementary material for: Clinician Perceptions of Family-Centered Care in Pediatric and Congenital Heart Settings
Source: JAMA Netw Open. 2024 Jul 15;7(7):e2422104. doi: 10.1001/jamanetworkopen.2024.22104 (PMC11250268; doi:10.1001/jamanetworkopen.2024.22104)
Supplement: Supplement. — Data Sharing Statement [file jamanetwopen-e2422104-s001.pdf]

Data Sharing Statement

Akram. Clinician Perceptions of Family-Centered Care in Pediatric and Congenital Heart Settings. *JAMA Netw Open*. Published July 15, 2024.
doi:10.1001/jamanetworkopen.2024.22104

Data
Data available: No

Additional Information
Explanation for why data not available: Data that underlie the results reported in this article may be available to suitably qualified researchers on request, after de-identification. Applicants willing to receive the data should apply between 1 and 12 months after the manuscript has been published and should demonstrate that the proposed use of the data has been approved by an independent review committee identified for this purpose. The data request should be sent to the corresponding author, nadine.kasparian@cchmc.org.
